# Emergence of KPC-134, a KPC-2 variant associated with ceftazidime-avibactam resistance in a ST11 *Klebsiella pneumoniae* clinical strain

**DOI:** 10.1128/spectrum.00725-23

**Published:** 2023-09-29

**Authors:** Xiangning Huang, Siquan Shen, Fan Chang, Xin Liu, Jinxi Yue, Ning Xie, Lin Yin, Fupin Hu, Daiwen Xiao

**Affiliations:** 1 Department of Laboratory Medicine and Sichuan Provincial Key Laboratory for Human Disease Gene Study, Sichuan Provincial People's Hospital, University of Electronic Science and Technology of China, Chengdu, China; 2 Institute of Antibiotics, Huashan Hospital, Fudan University, Shanghai, China; 3 Key Laboratory of Clinical Pharmacology of Antibiotics, Ministry of Health, Shanghai, China; 4 Department of Laboratory Medicine, Affiliated Hospital, North Sichuan Medical College, Nanchong, China; Emory University School of Medicine, Atlanta, Georgia, USA

**Keywords:** *Klebsiella pneumoniae*, ceftazidime-avibactam, KPC-134

## Abstract

The emergence of various new *Klebsiella pneumoniae* carbapenemase (KPC) variants leading to ceftazidime-avibactam treatment failure is a new challenge in current clinical anti-infection treatment. Here, we report a ceftazidime-avibactam-resistant *K. pneumoniae* 1072–2 clinical strain carrying a novel KPC variant, KPC-134, which differs from KPC-2 by both single mutation (D178A) and 8-amino acid insertions (asp-asp-asn-arg-ala-pro-asn-lys). The results of antimicrobial susceptibility testing showed that the isolate was resistant to meropenem (MIC = 4 mg/L), ceftazidime (MIC ≥ 32 mg/L), cefepime (MIC ≥128 mg/L), aztreonam (MIC ≥128 mg/L), and ceftazidime-avibactam (MIC ≥128 mg/L) but sensitive to imipenem (MIC = 0.5 mg/L), imepenem-relebactam (MIC = 0.5 mg/L), meropenem-vaborbactam (MIC = 2 mg/L), and aztreonam-avibactam (MIC = 4 mg/L). The plasmid containing *bla*_KPC-134_ was isolated from *K. pneumoniae*, and the *bla*_KPC-134_ gene was cloned into plasmid pHSG398 and transformed into an *Escherichia coli* DH5α to observe changes in antimicrobial resistance. The results indicated that the transformant was positive for *bla*_KPC-134_ and increased MICs of ceftazidime-avibactam, ceftazidime, cefepime, and aztreonam by 512-fold, 256-fold, 16-fold, and 4-fold, respectively, compared with the recipient. The results of third-generation sequencing showed that the *bla*_KPC-134_ gene was carried by a 133,789 bp IncFII-IncR plasmid, and many common resistance genes (including *bla*_CTX-M-65_, *bla*_TEM-1B_, *bla*_SHV-12_, *rmtB*, and *catB4*) along with the IS*26*, *tnpR*, *ISkpn8*, IS*kpn6*-like, and Tn*1721* elements were identified.

**IMPORTANCE:**

The emergence of various new KPC variants leading to ceftazidime-avibactam treatment failure is a new challenge for clinical anti-infection treatment. Here, we describe the characterization of a ceftazidime-avibactam-resistant blaKPC-134-positive *Klebsiella pneumoniae* clinical strain for the first time. *K. pneumoniae* bearing with KPC variant often mislead clinical anti-infection treatment because of their unique antimicrobial susceptibility profile and the tendency of conventional carbapenemase assays to give false negative results. Therefore, timely identification of KPC variants and effective anti-infective therapy are key to saving infected patients.

## OBSERVATION

In recent years, the widespread epidemic dissemination of carbapenem-resistant *Klebsiella pneumoniae* in the clinic has presented a major challenge for clinical anti-infective treatment (1, 2). According to the results of the China Antimicrobial Surveillance Network (www.chinets.com), the resistance rate of *K. pneumoniae* to imipenem or meropenem is rapidly increasing (from 3% in 2005 to more than 26% in 2022) (3). Available findings suggest that the production of carbapenemases, particularly class A KPC-type serine carbapenemases, is the predominant resistance mechanism for carbapenem resistance in *K. pneumoniae* (4). *In vitro* activities have shown that ceftazidime-avibactam has high antibacterial activity against KPC-type carbapenemase-producing *K. pneumoniae* and is the first-line drug for the clinical treatment of infections caused by such resistant bacteria (5 – 7).

However, with the widespread use of ceftazidime-avibactam in clinical practice, KPC-type carbapenemase-producing *K. pneumoniae* has mutated to adapt to the strong selective pressure of antibacterial drugs, and various new subtypes of KPC based on KPC-2 or KPC-3 mutations have emerged, mediating the resistance of *K. pneumoniae* to ceftazidime-avibactam and leading to treatment failure, which is currently a new challenge in clinical anti-infection treatment (8). The results of available studies show that the emergence of new subtypes of KPC genes has been an explosive trend in the last 3 years. To date, more than 180 *bla*_KPC_ subtypes have been reported in the world, according to the NCBI database (9). In this study, we describe the first characterization of *bla*_KPC-134_, a novel *bla*_KPC_ variant that confers resistance to ceftazidime-avibactam and restores susceptibility to imipenem.

The isolate *K. pneumoniae* 1072–2 was collected in a sputum sample from a 17-year-old female patient admitted to Sichuan Provincial People’s Hospital in 2022. The patient was admitted to the local hospital on 11 May 2022 with a recurrent fever for 7 d (maximum temperature of 40°C) and was diagnosed with pulmonary infection and peritonitis septicemia on admission. Sputum culture showed carbapenem-resistant *K. pneumoniae* 1072–1 (which is sensitive to ceftazidime-avibactam), and the anti-infective regimen included meropenem (1.0 g every 8 h for 19 d), tigecycline (50 mg every 12 h for 2 d), cefoperazone-sulbactam (1.5 g every 6 h for 11 d), caspofungin (50 mg every 24 h for 4 d), and ceftazidime-avibactam (2.5 g every 8 h for 14 d), but the treatment was not effective (Table 1). To seek further treatment, the patient was transferred to Sichuan Provincial People’s Hospital, and the anti-infective regimen was changed to imipenem (1 g every 8 h), amphotericin B (5 mg every 24 h), and polymyxin B (500,000 units q12h). The patient’s infection symptoms continued to progress, carbapenem-resistant *K. pneumoniae* continued to be cultured in sputum and ascites, and the patient eventually died from treatment failure. One ceftazidime-avibactam-resistant *K. pneumoniae* 1072–2 was isolated from sputum (Table 1)(10).

**TABLE 1:** Susceptibility of *K. pneumoniae* clinical isolate, transformant, and recipient to antimicrobial agents^*a*^

		MIC (mg/L)			
Antimicrobial agents	*K. pneumoniae* 1072–1(KPC-2)	*K. pneumoniae* 1072–2(KPC-134)	*E. coli* pHSG398-KPC-2	*E. coli* pHSG398-KPC-134	*E. coli* pHSG398
Imipenem	32(R)	0.5(S)	16(R)	0.25(S)	0.125(S)
Imepenem-relebactam	1(S)	0.5(S)	0.125(S)	0.125(S)	0.125(S)
Meropenem	>128(R)	4(R)	4(R)	≤0.06(S)	≤0.06(S)
Meropenem-vaborbactam	2(S)	2(S)	≤0.06(S)	≤0.06(S)	≤0.06(S)
Ceftazidime	>128(R)	>128(R)	16(R)	128(R)	0.5(S)
Ceftazidime-avibactam	8(S)	>128(R)	0.125(S)	64(R)	0.125(S)
Aztreonam	>128(R)	>128(R)	128(R)	0.5(S)	0.125(S)
Aztreonam-avibactam^*b*^	4(S)	4(S)	≤0.06(S)	≤0.06(S)	≤0.06(S)
Cefepime	>128(R)	>128(R)	4(SDD)	2(S)	0.125(S)
Amikacin	>128(R)	>128(R)	0.5(S)	0.5(S)	1(S)
Ciprofloxacin	>128(R)	>128(R)	≤0.06(S)	≤0.06(S)	≤0.06(S)
Polymyxin B	2(S)	0.5(S)	0.5(S)	0.5(S)	0.5(S)
Tigecycline	2(S)	2(S)	0.125(S)	0.125(S)	0.125(S)
Trimethoprim-sulfamethoxazole	>128(R)	>128(R)	0.125(S)	0.125(S)	0.125(S)

^*a*^ R, Resistant; S, susceptible.

^*b*^ Reference to the breakpoint of aztreonam (<=4 mg/L suscptible, 8 mg/L intermediate, and >=16 mg/L resistant).

PCR and DNA sequencing of the full-length *bla*_KPC_ gene revealed a novel *bla*_KPC_ variant, designated *bla*_KPC-134_. Nucleotide alignment of different *bla*_KPC_ variants showed that *bla*_KPC-134_ differs from *bla*_KPC-2_ by both single mutation (D178A) and 8-amino acid insertions (asp-asp-asn-arg-ala-pro-asn-lys). The plasmid containing *bla*_KPC-134_ was isolated from *K. pneumoniae*. In order to verify the function of the *bla*_KPC-134_ gene, we cloned the *bla*_KPC-134_ gene into the plasmid pHSG398 and transformed the recombined plasmid into *E. coli* DH5a to observe the change in antimicrobial resistance (Table 1). The transformant was positive for *bla*_KPC-134_ and increased MICs of ceftazidime-avibactam, ceftazidime, cefepime, and aztreonam by 512-fold, 256-fold, 16-fold, and 4-fold, respectively, compared with the empty vector-carrying strain (*E. coli*-pHSG398); however, there was essentially no change in MIC for imipenem and meropenem (Table 1).

**Fig 1:**
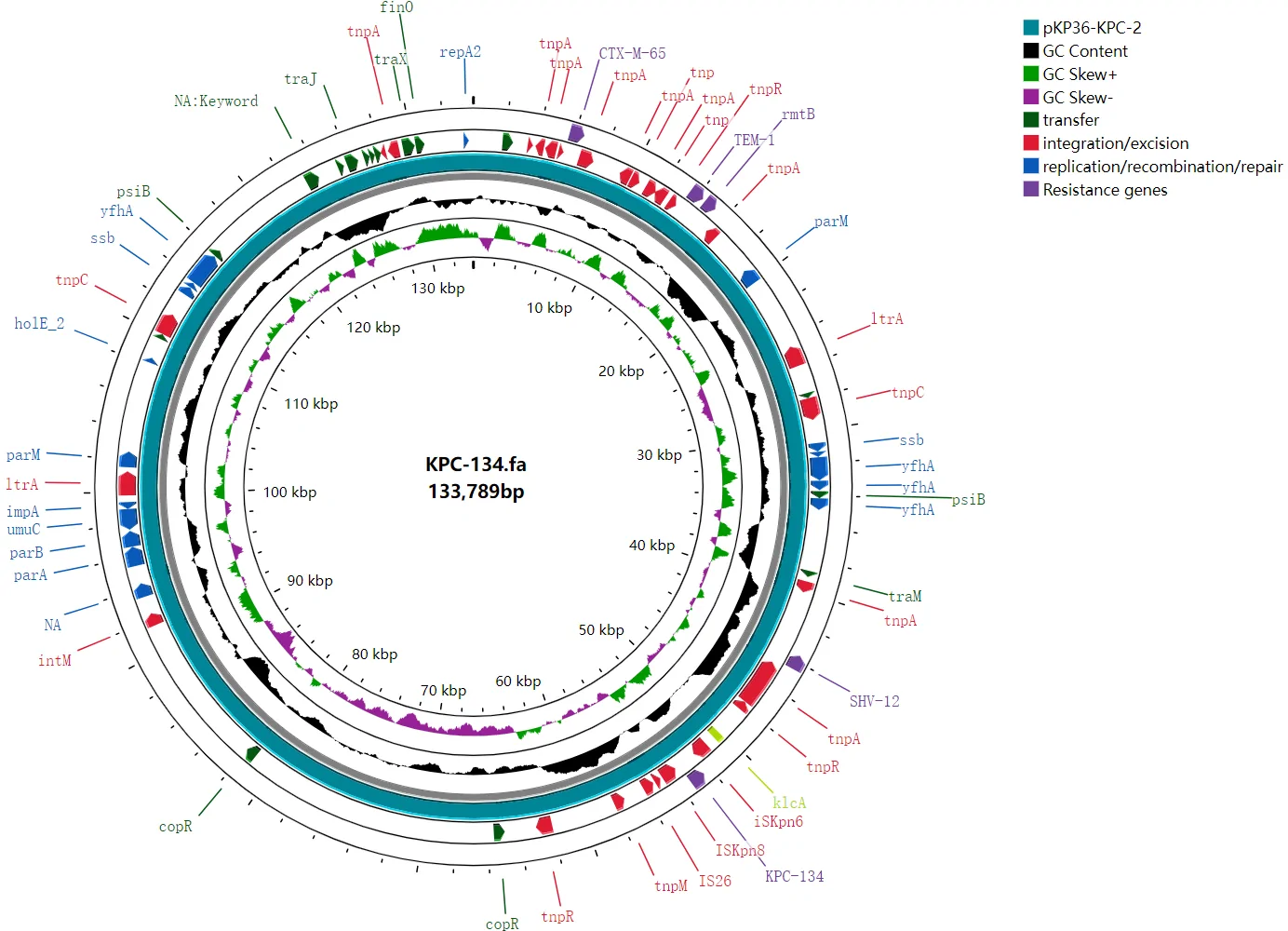
Alignments of plasmids. Comparison of the plasmids pKPC-134 and pKP36-KPC-2 using Proksee. A BLAST search for the sequence in GenBank showed that the sequence of pKP36-KPC-2-plasmid was very similar (99.95% coverage and 100% identity) to that of pKPC-134 (133,789 bp, GenBank accession no. OP293349.1), a plasmid of *K. pneumoniae* isolated from Sichuan, China.

**Fig 2:**
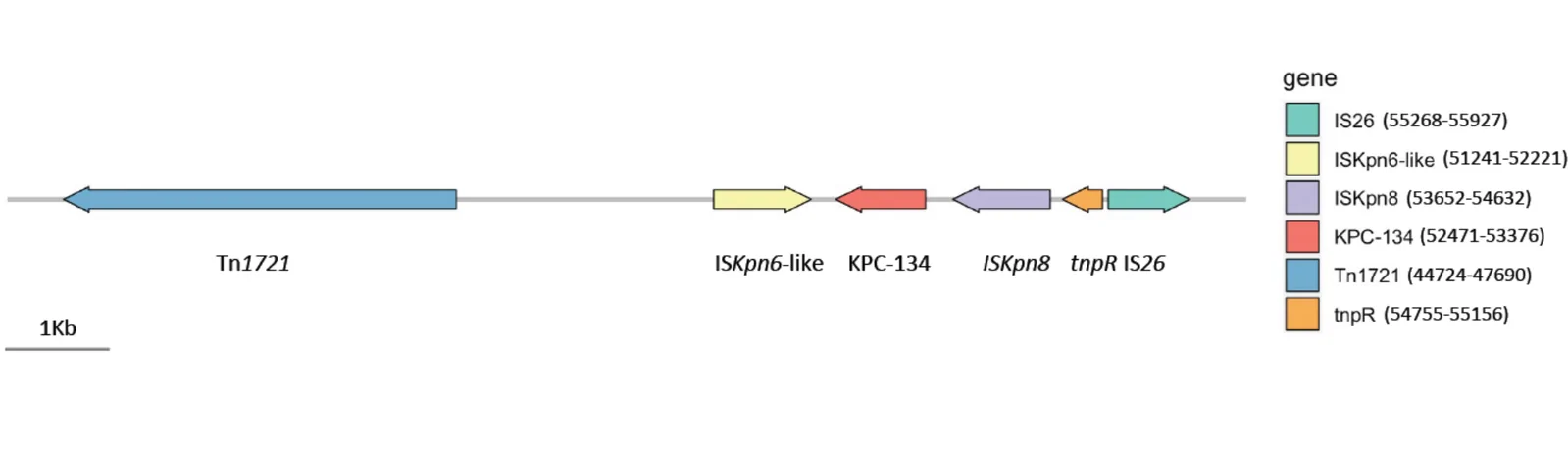
The genetic environment surrounding *bla*_KPC-134_. Genes, genetic elements, and other traits are color coded according to functional classification.

According to the whole-genome sequencing (WGS) analysis for the strain, many resistance genes had been identified, including the β-lactamase genes *bla*_KPC-134_, *bla*_CTX-M-65_, *bla*_TEM-1B_, and *bla*_SHV-12_, the aminoglycoside resistance genes *rmtB*, and the phenicol resistance gene *catB4*. Quinolone-related resistance gene mutations of GyrA (GyrA-83I and GyrA-87G) and ParC (ParC-80I) were found in *K. pneumoniae* 1072–2. According to the multilocus sequence typing result, strain *K. pneumoniae* 1072–2 belonged to ST11. The *bla*_KPC-134_ gene was carried by a 133,789 bp plasmid, which belonged to the IncFII-IncR type. The genetic structure of *bla*_KPC-134_ in pKPC-134 is identical to pKP36-KPC-2 (NZ_CP082761) (Fig. 1), carrying an IS*26*-based composite transposon, which is an 11.282 kb region including *bla*_KPC-2_ and one flanking IS*26* element; the complete genetic structure was IS*26*-tnpR-IS*Kpn8-bla*_KPC-134_-IS*Kpn6*-like-Tn*1721* (Fig. 2).

The emergence of various new KPC variants leading to ceftazidime-avibactam treatment failure is a new challenge for clinical anti-infection treatment (11). *K. pneumoniae* bearing with KPC-variant often mislead clinical anti-infection treatment because of their unique antimicrobial susceptibility profile and the tendency of conventional carbapenemase assays to give false negative results (12). Therefore, timely identification of KPC variants and effective anti-infective therapy are key to saving infected patients. The results of existing studies reported that the emergence of new subtypes of KPC in *K. pneumoniae* is overwhelmingly associated with the use of ceftazidime-avibactam (13, 14). Mutation derived from the *bla*_KPC-2_ or *bla*_KPC-3_ gene is the most reported resistance mechanism of *K. pneumoniae* to ceftazidime-avibactam, including amino acid deletions, mutations, insertions, and tandem repeats (15, 16). Compared with KPC-2, KPC-134 has both single mutation (D178A) and 8-amino acid insertions (asp-asp-asn-arg-ala-pro-asn-lys). It is worth noting that mutations within the Ω-loop that embrace the active site of KPC have been proven to enhance ceftazidime affinity and restrict avibactam binding (17 – 19). Interestingly, while mediating bacterial resistance to ceftazidime-avibactam, the new subtype of KPC tended to reduce the hydrolytic ability of the KPC enzyme to carbapenems, showing susceptibility to carbapenems, especially imipenem.

As reported, two major genetic structures, the Tn4401 transposon and the Tn3-Tn4401 transposon chimera, are mostly associated with KPC-2 (20, 21) and are considered the original genetic structure mediating *bla*_KPC_ gene acquisition worldwide (20, 22). In China, the genetic environment of the *bla*_KPC_ gene was distinct, with the Tn*1721-bla*_KPC_-IS*26* transposon chimera being the most common (23). The full genetic structure of *bla*_KPC-134_ in our study was IS*26*, *tnpR*, IS*Kpn8*, *bla*_KPC-134_, IS*Kpn6*-like element, and Tn*1721* transposase. It is known that the insertion sequence IS*26* can form a composite transposon, which can be excised from the plasmid to form a translocation unit (TU) (24). As mentioned above, the unique enzymatic hydrolysis characteristics and antimicrobial susceptibility profile of the new subtype of KPC raise a challenge for routine carbapenemase detection assays (12). KPC variants usually show false-negative results (12). The misleading detection in the clinical laboratory may lead to incorrect clinical decision-making and treatment failure. Therefore, the development of effective genetic testing methods for KPC variants and the enhancement of ceftazidime-avibactam susceptibility testing to better screen for KPC variants are ongoing efforts. In addition, studies have shown that meropenem-vaborbactam (25), for example, is considered a salvage therapy for the infection caused by ceftazidime-avibactam-resistant KPC variant-producing *K. pneumoniae*, but the new antimicrobial agent is not available in all countries or regions, especially in low-resource countries or regions. Therefore, for countries or regions lacking new antimicrobial agents, there is an urgent need to actively search for other alternative therapeutic options for the treatment of infections caused by KPC variant-producing strains, such as ceftazidime-avibactam in combination with imipenem (26).

## Data Availability

The genome sequencing data are publicly available at NCBI GenBank under the BioProject accession number OP293349.
